# In vitro and ex vivo protoscolicidal effect of poly(amidoamine) nanoemulsion against *Echinococcus granulosus*

**DOI:** 10.1038/s41598-024-62015-0

**Published:** 2024-05-25

**Authors:** Dina Aboelsoued, Nagwa I. Toaleb, Sally Ibrahim, Saber Ibrahim

**Affiliations:** 1 Department of Parasitology and Animal Diseases, Veterinary Research Institute, National Research Centre, Dokki, Giza, Egypt; 2 Department of Animal Reproduction and AI, Veterinary Research Institute, National Research Centre, El Buhouth Street, Dokki, Giza, Egypt; 3 Packaging Materials Department, National Research Centre, Dokki, Giza, 12622 Egypt; 4 Nanomaterials Investigation Lab., Central Laboratory Network, National Research Centre, Dokki, Giza, 12622 Egypt

**Keywords:** Poly(amidoamine), PAMAM, *Echinococcus granulosus*, Cystic echinococcosis, Protoscolices, SEM, In vitro, Ex vivo, Parasitology, Experimental models of disease

## Abstract

Hydatidosis causes a serious health hazard to humans and animals leading to significant economic and veterinary and public health concern worldwide. The present study aimed to evaluate the in vitro and ex vivo protoscolicidal effects of synthesized poly(amidoamine), PAMAM, nanoemulsion. In this study, PAMAM was characterized through dynamic light scattering technique to investigate the particle size and zeta potential of nanoemulsified polymer. For the in vitro and ex vivo assays, we used eosin dye exclusion test and scanning electron microscope (SEM) to evaluate the effects of the prepared and characterized PAMAM nanoemulsion against protoscoleces from *Echinococcus granulosus *sensu lato G6 (GenBank: OQ443068.1) isolated from livers of naturally infected camels. Various concentrations (0.5, 1, 1.5 and 2 mg/mL) of PAMAM nanoemulsion at different exposure times (5, 10, 20 and 30 min) were tested against protoscolices. Our findings showed that PAMAM nanoemulsion had considerable concentration- and time-dependent protoscolicidal effect at both in vitro and ex vivo experiments. Regarding in vitro assay, PAMAM nanoemulsion had a potent protoscolicidal effect when compared with the control group with a highest protoscolicidal activity observed at the concentration of 2 mg/mL at all exposure times, such that 100% of protoscolices were killed after 20 min of exposure. Also, the mortality of protoscolices was 100% after 30 min of exposure to 1 and 1.5 mg/mL of PAMAM nanoemulsion, in vitro. Concerning ex vivo assay PAMAM nanoemulsion recorded the highest mortality rates at the concentration of 2 mg/mL (55, 99.4 and 100% at 10, 20, 30 min, respectively). Ultrastructure examination of examined protoscolices after 20 min of exposure to PAMAM nanoemulsion showed a complete loss of rostellar hooks, disruption of suckers with disorganization of hooks with partial or complete loss of them, and damage of protoscolices tegument with loss of their integrity in the form of holes and contraction of the soma region were observed in 1.5 and 2 mg/mL of PAMAM, in vitro and ex vivo*,* showing more damage in the in vitro conditions. It can be concluded that PAMAM nanoemulsion is a promising protoscolicidal agent offering a high protoscolicidal effect at a short exposure time. Further in vivo studies and preclinical animal trials are required to evaluate its efficacy and clinical applications against hydatid cysts.

## Introduction

Hydatidosis or cystic echinococcosis (CE) occurs accidentally with the eggs of *Echinococcus granulosus*, shed with stool of the definitive host (carnivores such as: canines), in human and animal intermediate hosts such as: buffaloes, cattle, sheep, goats, camels, etc*.*, followed by the development of the larvae^1,2^. This disease is characterized by the long-lasting growth of fluid-filled hydatid cysts spread in the viscera of intermediate hosts^3^. It can cause a serious health threat to humans and animals, regarding the stage and location of the cyst, resulting in a significant veterinary and medical importance with economic and public health concern worldwide^4^. Hydatidosis effect on livestock industry appears in the form of condemnation of the infected meat and organs, retarded growth, decreased fertility, weight loss, decreased milk production, increased mortality, decreased hide value and fecundity^5,6^.

*Echinococcus granulosus* is the most prominent species due to its worldwide distribution, with high occurrence in domestic animals with pastoral activities and humans^7^. In animals, it is asymptomatic, and its diagnosis is performed at necropsy while post-mortem inspection^8^. In this disease, different pathological lesions and abnormal organ tissues are found in slaughtered animals which were apparently healthy^9,10^. The control of hydatidosis requires a good understanding of all aspects related to the parasite survival, particularly protoscolex^11^ and it is achieved by long term prevention strategies targeting meat inspection, dogs’ deworming, health education, and surveillance in livestock and human individuals^12^. Its treatment depends on the stage, location, size, and complications of the hydatid cysts^13^. Many single or combined treatment trials were conducted against hydatidosis^14,15^. In humans, surgery is the preferred option for treatment of cystic echinococcosis; however, current protoscolicidal treatments used during surgery have many side effects and complications^16,17^. The most important risk of cystic echinococcosis surgical approaches is the rupture of cysts and/or leakage of their contents, which could cause re-infection, anaphylaxis shock, and even death in some cases patients^18^.

Due to many advantages of being safe, non-toxic, clean, and environmentally friendly with minor side effects, the synthesis of nanoparticles (NPs) has gained attention and popularity due to their superior activities in pharmacological applications^19,20^. In previous studies, poly(amidoamine) (PAMAM) nanoemulsion showed many biomedical applications^21^ such as antimicrobial^22–26^ and antiparasitic^27–30^ activities. Also, PAMAM is a synthetic polymer having various biological properties, being non-toxic, good water solubility, highly biocompatible, and biodegradable^31,32^. Interestingly, PAMAM dendrimer is widely used as an analog to enzymes, proteins, viruses, and other bioactive agents as it is primarily focusing the target cells and conjugating to host cells^33^. Also, it can be used as DNA and gene carrier, in drug delivery systems and tissue engineering^32,34^.

Finding a safe and effective alternative protoscolicidal agent for the treatment of cystic echinococcosis in humans and animals is urgently required. Accordingly, the present study aimed to evaluate the protoscolicidal effects of PAMAM nanoemulsion both in vitro and ex vivo.

## Materials and methods

### PAMAM NPs

The product (DENTRIC-GEN 002) as biocompatible, biodegradable, nanopolymeric material was supplied from Nanomaterials Investigation Laboratory, Central Laboratory Network, National Research Centre, Egypt.

#### Characterization of DENTRIC-GEN 002 NPs

The particle size and zeta potential of nanopolymeric DENTRIC-GEN 002 were analyzed using NICOMP 380 ZLS, PSS, Santa Barbara, CA, USA. The particle size was measured according to dynamic light scattering with 700 measurements of collision laser beam with DENTRIC-GEN 002 particles^35^. Zeta potential as particle charge through applied electrical current on alloy electrodes^36^.

### Parasite

#### Parasite isolate

We used the *E. granulosus *sensu lato G6 (GenBank: OQ443068.1) previously isolated from livers of naturally infected camels slaughtered at Nahia abattoir, Giza (29° 58′ 27.00″ N, 31° 08′ 2.21″ E), Egypt, and identified using NADH dehydrogenase subunit 1 (NAD1) gene by PCR^37^.

#### Protoscolices preparation

The hydatid cyst fluid was aseptically aspirated, transferred into a glass container and left to settle down for 30 min. Then the settled down protoscolices were centrifuged at 800 rpm for 10 min^38^. The supernatant was discarded and the sedimented protoscolices were collected in one tube. Viability of protoscolices was evaluated using the eosin exclusion test in which 10 μL of 0.1% aqueous Eosin stain (Sigma-Aldrich, Louis, MO, USA) was mixed with 10 μL of protoscolices pellet and examined under a low-power microscope (CX41 Olympus Microscope, Olympus Corporation, Japan) after 5 min as described by Daryani et al.^39^. After 5 min of exposure, the unstained protoscolices were deemed viable, while stained protoscolices were considered dead. When 95% or more viable protoscolices were recorded in the sediments, then the sample was appropriate for the experiments^40^.

#### In vitro protoscolicidal assay

Different concentrations of PAMAM nanoemulsion including 0.5, 1, 1.5 and 2 mg/mL were used for different exposure times which were: 5, 10, 20, 30 min^41,42^. Protoscolices tubes of control untreated groups, for each incubation time, were treated by normal saline only. Thereafter, for each experiment, 2 mL of the solution was placed in a small test tube. Then one drop of the protoscolices-rich sediment containing about 1000–1500 protoscolices was added to each tube using Pasteur pipettes. After mixing the contents, the tubes were incubated at 37 °C for 5, 10, 20, 30 min. The experiments were performed in triplicates.

#### Ex vivo protoscolicidal assay

Fertile liver hydatid cysts were acquired from naturally infected camels slaughtered at Nahia abattoir, Giza (29° 58′ 27.00″ N, 31° 08′ 2.21″ E), washed by phosphate buffered saline (PBS) and used. Firstly, about 50% of each cyst contents were aspirated to determine the viability of protoscolices as described before. Same concentrations of PAMAM nanoemulsion used in ex vivo experiment including 0.5, 1, 1.5 and 2 mg/mL for different exposure times which were: 5, 10, 20, 30 min. For each concentration, three hydatid cysts were used and PAMAM nanoemulsion were injected into each cyst.

#### Viability test after treatment

To assess the viability of protoscolices at the end of each incubation period, the upper part of each tube was carefully discarded and 1 mL of 0.1% eosin stain solution was added and mixed gently^43^. For ex vivo experiment, small amount of cyst fluid along with protoscolices was aspirated in a tube and 0.1% eosin was added. The stained protoscolices were smeared on a glass slide, then covered with a cover glass, and examined under light microscope (CX41 Olympus Microscope, Olympus Corporation, Japan). Percentage of mortality rate was estimated by counting 100 protoscolices using eosin exclusion test^44^. Dyed protoscolices were recorded as dead, protoscolices that didn’t absorb stain were considered as viable^45^.

#### Ultrastructural examination of protoscolices

Ultrastructural examination using Scanning Electron Microscope (SEM) was conducted. Protoscolices under experiment were fixed in 2.5% glutaraldehyde for 2 h at room temperature, fixed in 100 mM sodium cacodylate buffer (pH 7.2) for 2 h at room temperature, processed for SEM^46^ and examined by a QUANTA FEG 250 (USA) SEM operating system.

### Statistical analysis

Data were analyzed using SPSS 20 (IBM-SPSS, Inc., Chicago, IL, USA). The results of protoscolicidal effect experiments were analyzed using One-way analysis of variance (ANOVA) and Duncan’s test, then were expressed in percentages. *P* < 0.05 was considered significant.

### Ethics approval

All experimental procedures were conducted according to the National Research Centre’s Animal Research Committee institutional guidelines the under protocol number: 2180212023.

## Results

### Characterization of PAMAM nanoemulsion

**Figure 1:**
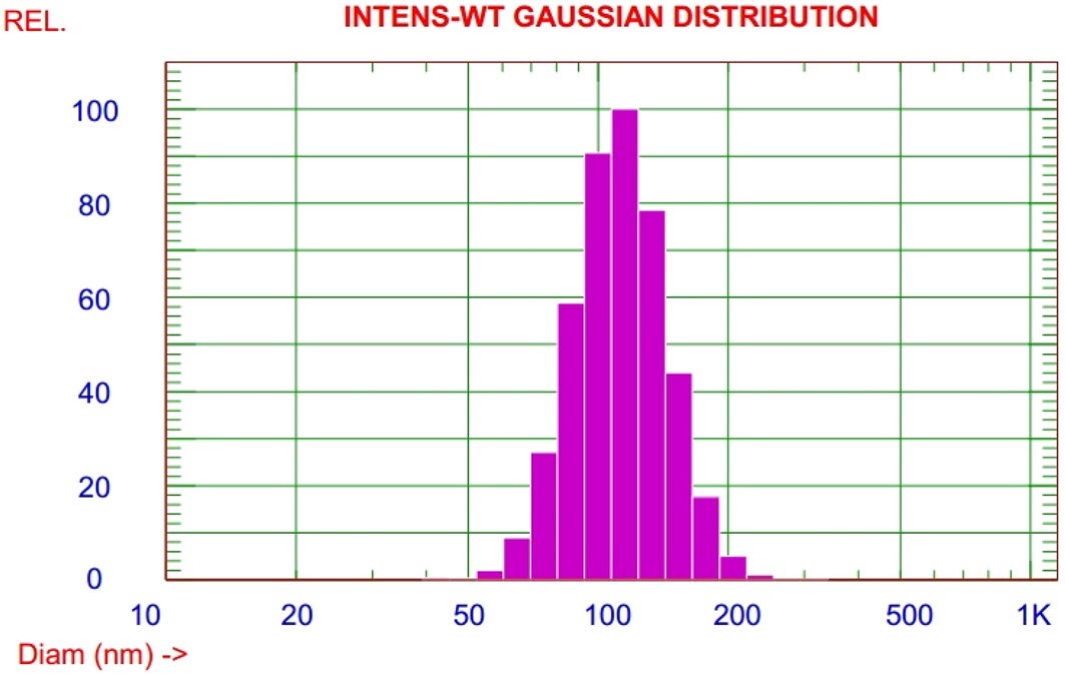
Particle size distribution of PAMAM nanoemulsificaion biocompatible dendrimer polymer by DLS.

**Figure 2:**
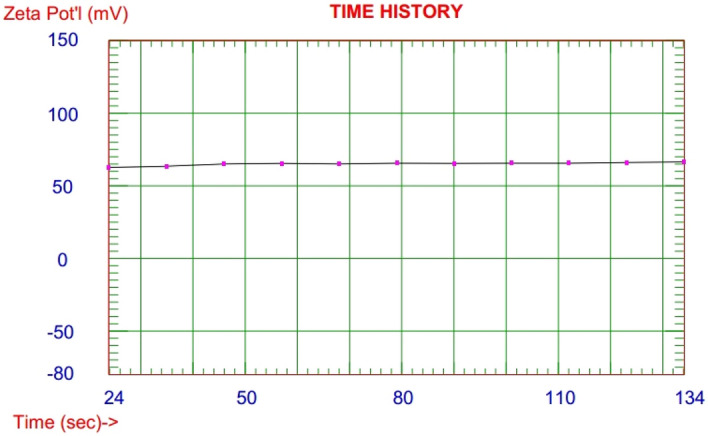
Zeta potential of PAMAM nanoemulsificaion biocompatible dendrimer polymer by DLS.

Figures 1 and 2: illustrated homogenized particle size bell shape distribution with narrow poly dispersity index 0.061 and mean particle size 115.5 nm. Where the zeta potential measurements show stable value over eleven cycles of application electric current with average zeta potential + 66.36 mV.

### Viability test

**Figure 3:**
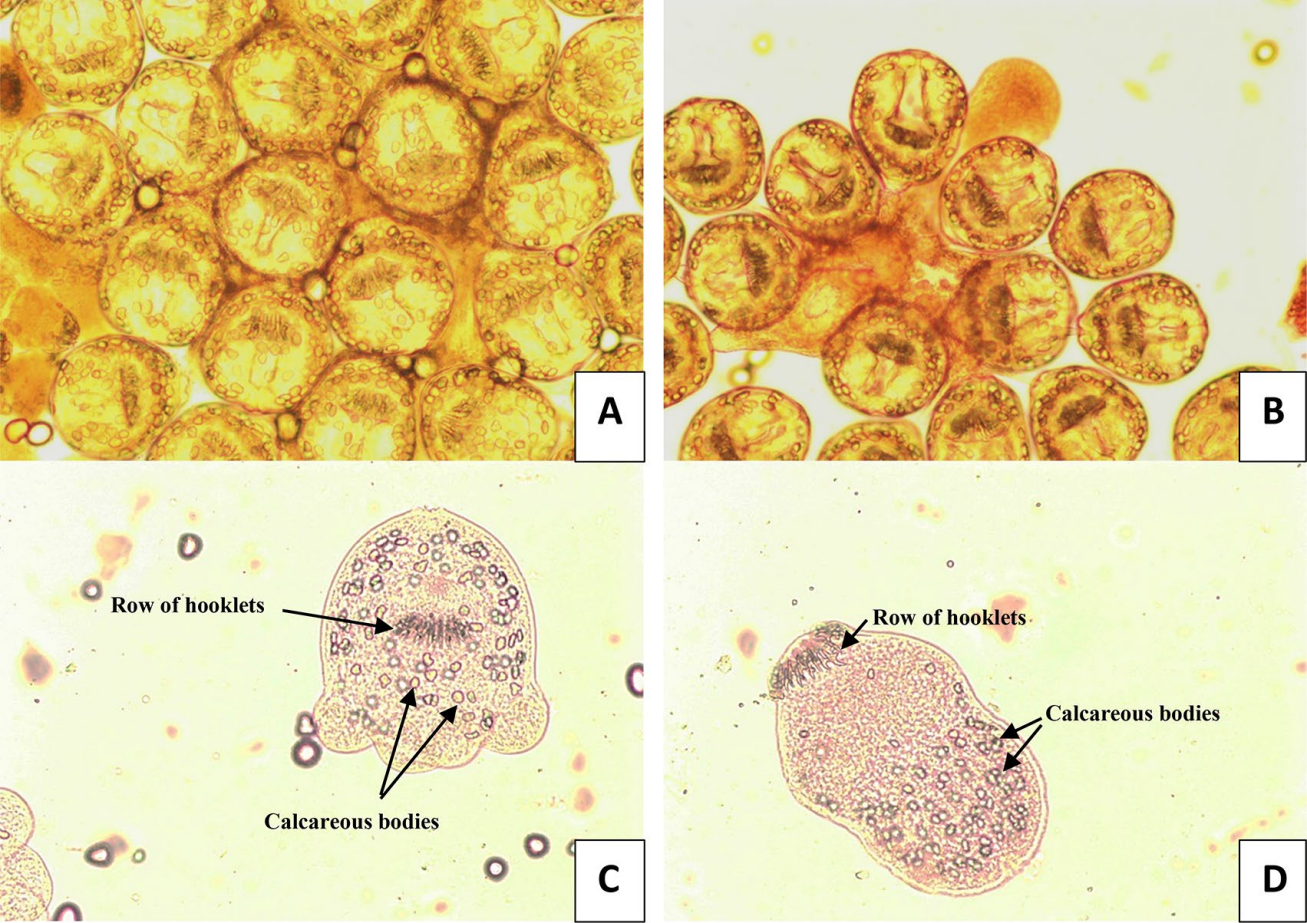
Light microscopy images showing the viable protoscolices of *Echinococcus granulosus* before assays (**A** & **B**: × 100). Invaginated protoscolex (**C**) and Evaginated protoscolex (**D**) showing rostellum, hooklets and calcareous bodies (**C** & **D**: × 400).

The collected protoscolices were examined by light microscopy for their viability with eosin exclusion test. Results showed that about 97% were viable and rostellum, hooks and calcareous bodies were visible (Fig. 3A–D).

### Protoscolicidal effect of PAMAM nanoemulsion

#### In vitro protoscolicidal effect of PAMAM nanoemulsion at different concentrations and exposure times

**Figure 4:**
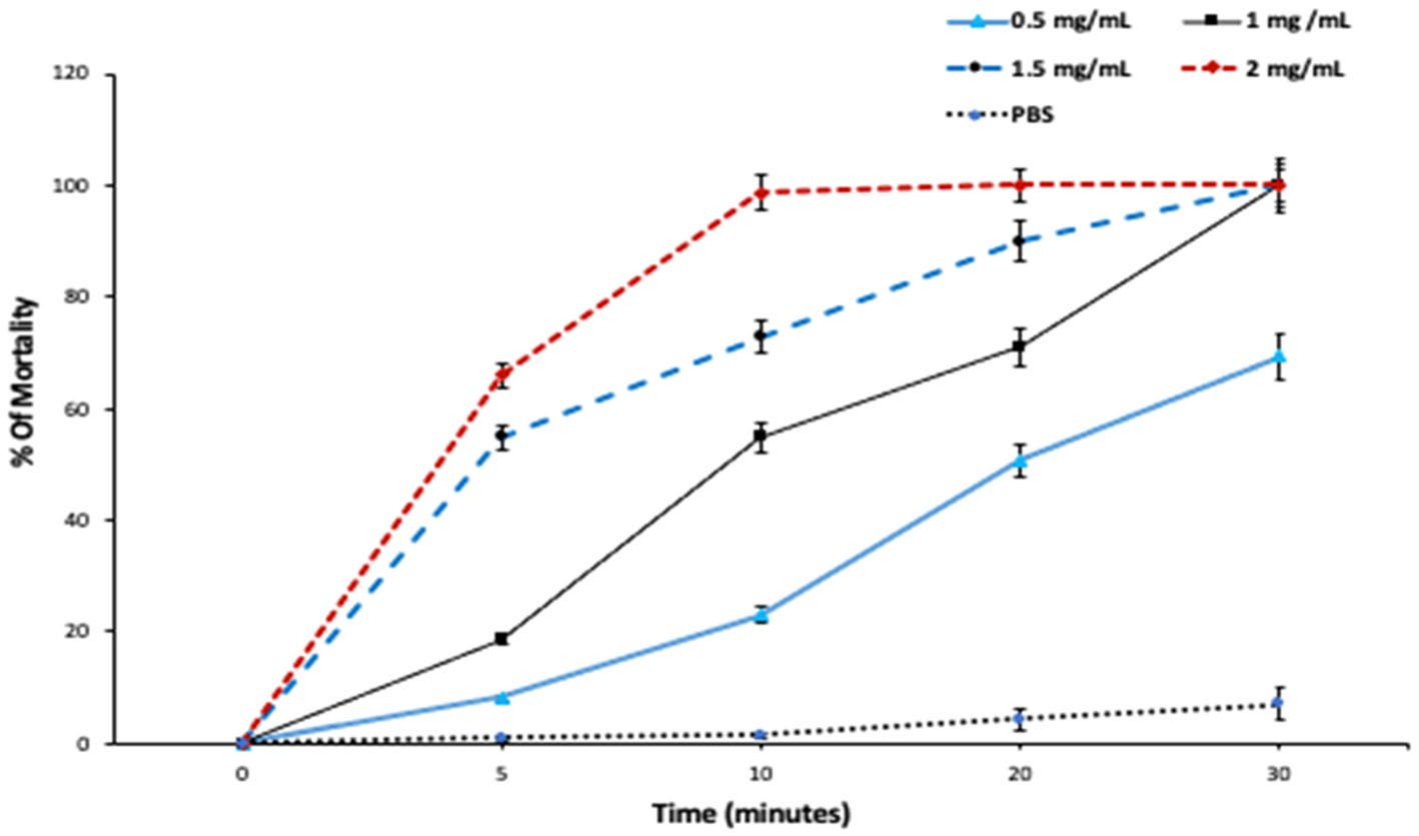
In vitro protoscolicidal effect of PAMAM nanoemulsion at different concentrations and exposure times.

**Figure 5:**
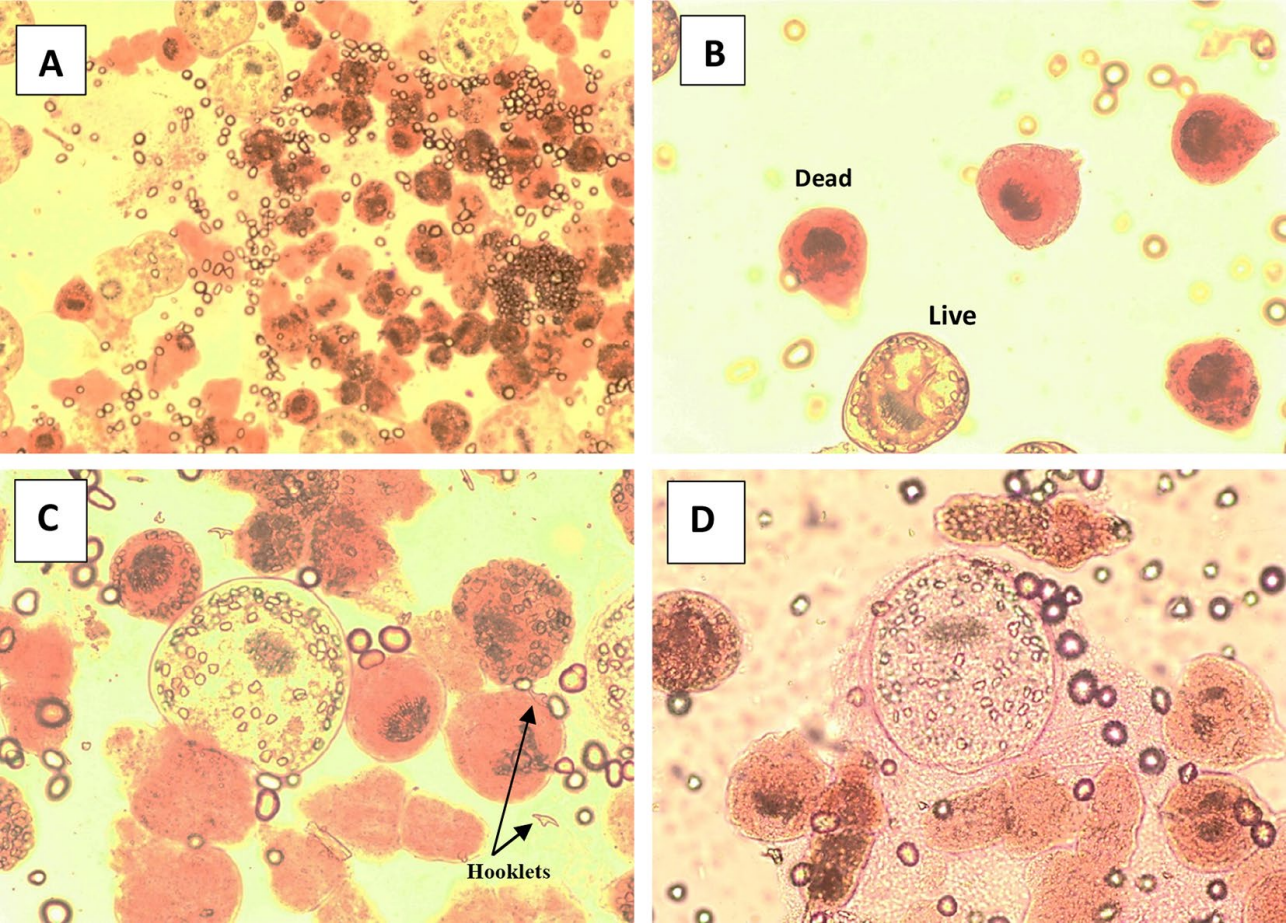
Live and red stained dead protoscolices after exposure to PAMAM nanoemulsion (1.5 mg/mL) after 20 min of exposure (stained with 0.1% Eosin), (**A**: × 40, **B**: × 100, **C** & **D**: × 400).

Regarding in vitro antiparasitic effect of the different concentrations of PAMAM nanoemulsion on the *E. granulosus* protoscolices after 5-, 10-, 20-, and 30-min incubation, it was found that PAMAM nanoemulsion had considerable significant (*P* < 0.001) protoscolicidal effects when compared with the control group (Figs. 4 and 5A–D). The findings exhibited that a highest protoscolicidal activity (*P* < 0.001) was observed at the concentration of 2 mg/mL at all exposure times, such that 100% of protoscolices were killed after 20 min of exposure. Also, the mortality of protoscolices was 100% after 30 min of exposure to 1 and 1.5 mg/mL. The mortality rate of protoscolices in the control group (PBS) was 7.1% at the end of experiment, after 30 min of exposure.

#### Ex vivo protoscolicidal effect of PAMAM nanoemulsion at different concentrations and exposure times

**Figure 6:**
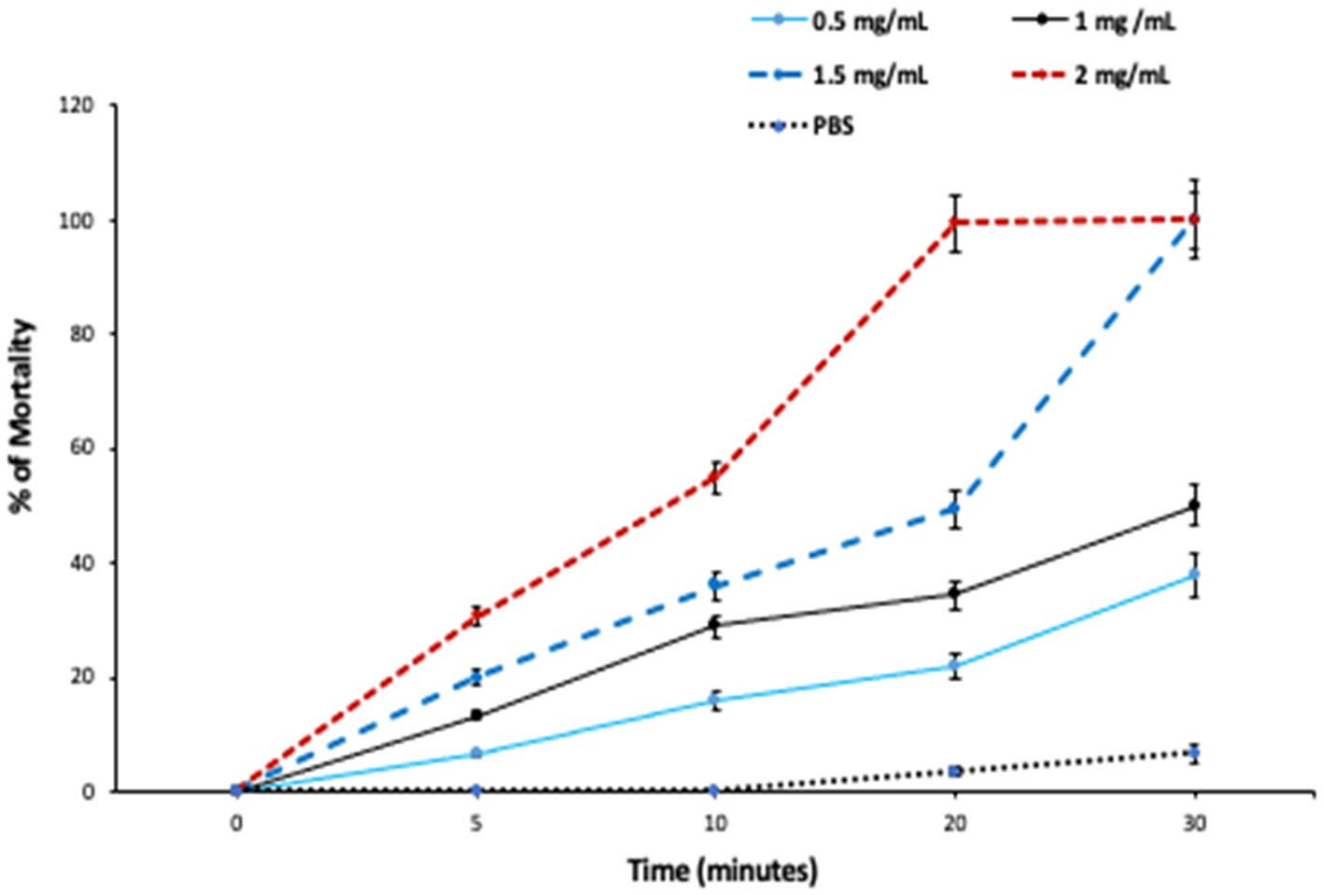
Ex vivo protoscolicidal effect of PAMAM nanoemulsion at different concentrations and exposure times.

Our obtained results indicated that PAMAM nanoemulsion had a remarkable concentration- and time-dependent protoscolicidal effect ex vivo when compared with the control group recording the highest significant mortality rates at the concentration of 2 mg/mL (30.6, 55, 99.4 and 100% at 5, 10, 20, 30 min, respectively, *P* < 0.001). It’s worth mentioning that PAMAM nanoemulsion required longer time to kill protoscolices in ex vivo conditions (Fig. 6).

### Ultrastructural examination of protoscolices

**Figure 7:**
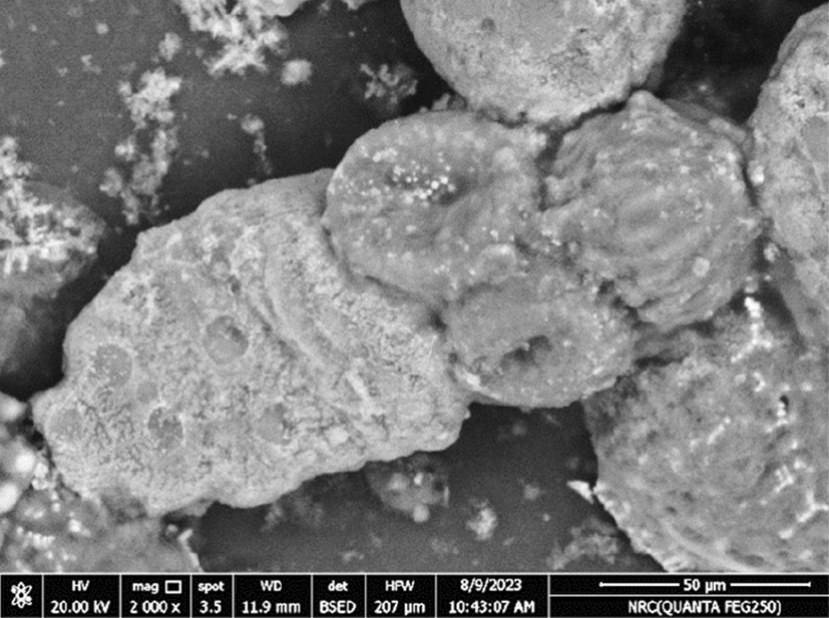
Scanning electron microscopy of *Echinococcus granulosus* protoscolex incubated with PBS (× 2000).

**Figure 8:**
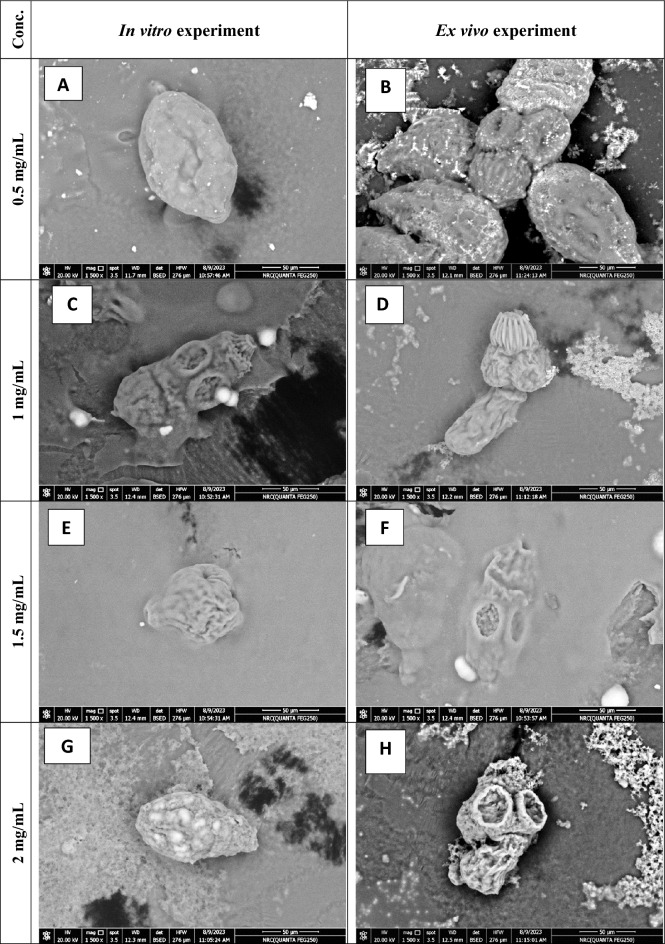
Scanning electron microscopy of PAMAM nanoemulsion-treated *Echinococcus granulosus* protoscolices with different concentrations at 20 min of incubation. Electron micrographs show concentration-dependent effect of PAMAM nanoemulsion on *E. granulosus* protoscolice*s *in vitro and ex vivo (× 1500 **A**–**H**).

Figure 7 shows evaginated control *E*. *granulosus* protoscolex (× 2000). Figure 8A–H shows altered protoscolices after 20 min incubation with concentrations of 0.5, 1, 1.5 and 2 mg/mL of PAMAM nanoemulsion (× 1500). Electron micrographs show concentration-dependent effect of PAMAM nanoemulsion on *E. granulosus* protoscolices in vitro and ex vivo*.* A complete loss of rostellar hooks and disruption of suckers are observed in PAMAM concentrations 1.5 and 2 mg/mL in vitro. The protoscolices tegument were markedly damaged with loss of their integrity in the form of holes and contraction of the soma region. Suckers appeared disrupted with disorganization of hooks with partial or complete loss of them.

## Discussion

CE, the parasitic larval cyst stage of the tapeworm *E. granulosus*, has become a threat to global public and animal health. Chemical compounds targeting CE which are recommended by the World Health Organization (WHO) are mebendazole and albendazole, but they showed poor efficacy with low dissolution rate, poor absorptivity^47^, several side-effects and drug resistance under long-term treatment^48^. Researchers worldwide are trying to uncover novel compounds are urgently needed for the treatment of this disease. Nanomedicine is a well-known field of science which uses nano-meter-sized materials for various medical goals such as: diagnosis and therapy of many diseases, drug delivery, tissue engineering, imaging, biological protection, vaccines, etc*.*^49,50^. Leakage of protoscoleces may happen during surgical operations and also CE recurrence rates were reported to be 10–30% and 2–12% after surgical treatment of hepatic and pulmonary CE, respectively. So, scolicidal agents have an important role in treatment and prevention of secondary hydatid disease^44^. In the current study, we used PAMAM nanoemulsion which may be capable of treating CE as PAMAM has a wide range of biomedical applications due to its physicochemical properties^21,28,29,51^. in our study, The particle size distribution and zeta potential of PAMAM nanoemulsificaion biocompatible dendrimer polymer was performed and our results indicated good particle size distribution in nanometer range of PAMAM with cationic positive charge zeta potential with high value that point to excellent stability of nanoemulsified polymer. Excellent structural properties and good water solubility of PAMAM make it a good candidate material for bio-applications^31,32,51^.

In our study, the collected protoscolices were examined for their viability with eosin exclusion test. Results of protoscolices viability showed that about 97% were viable with arranged hooks, uniform tegument and visible rostellum and calcareous bodies. Similar observations were recorded by Toaleb et al.^37^, Barabadi et al.^52^ and Fateh et al.^53^.

Developing alternative protoscolicidal agents and nano-preparations with lower side effects and higher potency is necessary^54^. Regarding in vitro and ex vivo protoscolicidal effect of the different concentrations of PAMAM nanoemulsion after different incubation times, we found that PAMAM nanoemulsion had considerable concentration- and time-dependent protoscolicidal effect. These results might be attributed to the activity of PAMAM. In other studies, amine terminated PAMAM showed antibacterial activity^22–26^ and induced cell death in *Trypanosoma cruzi* epimastigotes via apoptosis^29^. PAMAM uniform branching offers a large surface area to volume ratio which enables high reactivity with microorganisms in vivo^55^. Ideal protoscolicidal agents should be nontoxic, easy prepared, potent at low concentration and within a short period of time, able to kill the scolex and daughter cyst inside a cyst and stable in the presence of cyst fluid^56^.

Metal NPs and metal oxide NPs are the most widely utilized NPs for the treatment hydatid cyst followed by polymeric NPs^57^. Iron, silica, silver, copper, and zinc NPs^41^, gold NPs^42,52^, copper NPs^17^, silver NPs^38,58,59^, zinc oxide NPs^60^ showed less protoscolicidal activities than PAMAM^61^. Malekifard et al.^62^ tested the effect of gold NPs on *E. granulosus* scolices and found that all concentrations of gold NPs had significant scolicidal effect and killed all protoscolices at 60 min at the concentration of 1 mg/ml. Also, Norouzi et al.^41^ observed that the highest scolicidal effect (80%) of silver NPs at the concentration of 1 mg/mL after 60 min. Differences recorded in our results and other studies might be due to the differences in the type of NPs, concentration, exposure time^63^, surface properties, adsorption, and penetration of the NPs^64^.

Since 60 min exposure is a relatively long time for application during surgical operations, PAMAM nanoemulsion prepared in the present study showed better effect at shorter incubation time as it recorded 72.7, 90 and 100% mortality rate in 10, 20, 30 min, respectively, with the concentration of 1.5 mg/mL in vitro and 55, 99.4 and 100% protoscolices mortality rate in 10, 20, 30 min, respectively, with the concentration of 2 mg/mL ex vivo. We also noted that PAMAM nanoemulsion required longer time to kill protoscolices in ex vivo conditions. This observation was also reported by Niazi et al.^65^, Mahmoudvand et al.^66,67^ and Raziani et al.^68^ indicating that the protoscolicidal agents require a further incubation time to show a potent effect ex vivo.

In the present study, ultrastructural examination of protoscolices after 20 min incubation with concentrations of 0.5, 1, 1.5 and 2 mg/mL of PAMAM nanoemulsion in vitro and ex vivo showed the alterations in protoscolices’ tegumental damage, loss of tegument integrity, holes and contraction of the soma region, disruption of suckers and disorganization of hooks with partial or complete loss of them. Similar findings were recorded using NPs, essential oils, synthetic drugs, medicinal plants, and plant-derived compounds^41,68–71^. The efficacy of PAMAM nanoemulsion, used in our study, might be subjected to the protoscolices’ tegument, which is one of early sites of damage, as the loss of protoscolices’ tegumental integrity could be related to apoptosis. Thus, it might prevent establishment of cyst by altering membrane integrity and hinder the formation of a new cyst or a secondary CE^69^. In our study, nanoemulsion delivery system for PAMAM could improve the efficacy and bioavailability of its active components by their solubilization into nanodroplets^72^. As a result, it might enhance the interaction of these components with the protoscolices membranes by increasing the surface area^73^ and the thermodynamic stability of the nanoemulsion could provide a sustained release of PAMAM at the targeted site of action by the fusion of nanodroplets with the phospholipid layer of the cell membrane which enhances its activity^74,75^.

## Conclusion

The current study describes the in vitro and ex vivo effect of PAMAM nanoemulsion against protoscolices of *E. granulosus*. In our study, PAMAM exhibited excellent particle size distribution with high zeta potential as stable dispersed nanopolymeric materials in aqueous medium. Also, the results showed a time-dependent effect provoking the loss of protoscolex viability. Based on our findings, it can be concluded that PAMAM nanoemulsion is a promising and potent protoscolicidal agent offering a high protoscolicidal effect at a short exposure time. However, further in vivo studies and preclinical animal trials are required to evaluate the efficacy of PAMAM nanoemulsion and its clinical applications against hydatid cysts.

## Data Availability

The datasets used and/or analyzed during the current study available from the corresponding author on reasonable request.
